# Identifying Choroidal Neovascularization-Associated Genes with GenePlexus in a Protein–Protein Interaction Network

**DOI:** 10.3390/life16081263

**Published:** 2026-07-30

**Authors:** Lihua Liu, Yuhang Zhang, Feiming Huang, Yusheng Bao, Jian Zhang

**Affiliations:** 1 Department of Ophthalmology, Shanghai Yangzhi Rehabilitation Hospital (Shanghai Sunshine Rehabilitation Center), School of Medicine, Tongji University, Shanghai 201619, China; 2 Channing Division of Network Medicine, Brigham and Women’s Hospital, Harvard Medical School, Boston, MA 02115, USA; reyhz@channing.harvard.edu; 3 School of Life Sciences, Shanghai University, Shanghai 200444, China; hfm123@shu.edu.cn (F.H.); bys@shu.edu.cn (Y.B.); 4 Department of Ophthalmology, Shanghai General Hospital, Shanghai Jiao Tong University School of Medicine, Shanghai 200080, China; 5 National Clinical Research Center for Eye Diseases, Shanghai 200080, China; 6 Shanghai Key Laboratory of Ocular Fundus Diseases, Shanghai 200080, China; 7 Shanghai Municipal Hospital Ophthalmology Specialist Alliance, Shanghai 200080, China; 8 Shanghai Engineering Center for Visual Science and Photomedicine, Shanghai 200080, China; 9 Shanghai Engineering Center for Precise Diagnosis and Treatment of Eye Diseases, Shanghai 200080, China

**Keywords:** choroidal neovascularization, disease gene, protein–protein interaction, GenePlexus

## Abstract

Choroidal neovascularization (CNV) is a common and serious complication in various retinal diseases that involves the growth of new blood vessels from the choroid into the subretinal space, leading to a visual threat. Its underlying mechanism has not been fully uncovered. Discovering new genes related to CNV is an important way to reveal its molecular mechanisms. In this study, we used DisGeNET as the initial data source to identify genes related to CNV. The gene cluster method and additional screening tests were designed to explore the genetic landscape related to CNV. Our comprehensive analysis revealed many genes with high confidence, specifically identifying novel candidates that traditional topological methods missed. Notably, we identified SERPINE1, COL1A1, and ANGPT1 as unique gene signatures using our network embedding approach. Functionally, SERPINE1 regulates the plasminogen activation system to control proteolytic balance, COL1A1 maintains the structural integrity of the extracellular matrix during invasion, and ANGPT1 is critical for the maturation and stabilization of nascent vessels. Meanwhile, some genes were also identified by other methods, such as MMP2, a regulator of extracellular matrix degradation. The newly found genes are essential for understanding the process that initiates CNV.

## 1. Introduction

The blood supply system is essential for maintaining the function of organs like the brain, lung, heart, and eyes [1,2,3]. Dysfunction of the blood supply system, such as pathogenic alterations involving blood vessels, disrupts vascular regulation balance [4]. Aging is one of the most common factors affecting the blood supply system throughout the entire body [5,6]. Specifically for the ocular system, overall pathological age-related changes are summarized as wet or exudative age-related macular degeneration (AMD) [7]. Choroidal neovascularization (CNV) is the main pathological process for pathological age-related changes, which is characterized by retinal epithelium tissue intravasation [8,9]. Patients with pathologic myopia have been reported to be more sensitive to choroidal neovascularization [10]. More than 5% of these patients will develop choroidal neovascularization, among whom patients with a history in one eye have more than a 30% probability of developing the pathology in the other eye [10], making this disease a severe threat to human health.

Current clinical nomenclature distinguishes CNV, macular neovascularization (MNV), and neovascular AMD (nAMD) [11]. MNV is an anatomical umbrella term for neovascular lesions in the macular region. Type 1 MNV is located beneath the retinal pigment epithelium, and type 2 MNV extends into the subretinal space; these lesions are generally of choroidal origin. By contrast, type 3 MNV originates from the retinal circulation and therefore should not be considered synonymous with CNV. nAMD is a disease context in which any of these MNV subtypes may occur, whereas CNV can also arise in pathologic myopia, inflammatory chorioretinopathies, and other disorders. Lesion type is clinically relevant because anatomical origin and treatment outcomes may differ among MNV subtypes [12]. The molecular mechanisms of CNV have been widely reported. Three major biological and pathological components contribute to the initiation and progression of CNV from different perspectives: (1) Neovascularization regulation factors, including VEGF, SDF-1, and their upstream regulator HIF-1. In 2008, researchers from the University of Liverpool revealed the associations between the SDF-1 alpha subunit and the VEGF signaling pathway, validating their specific roles during the pathogenesis of choroidal neovascularization [13]. A follow-up study in 2014 further linked the VEGF–SDF1 association with the TGF-beta signaling pathway [14], providing new therapeutic targets for CNV. (2) Tie2 pathway-activating signals from surrounding cells and the extracellular matrix influence vascular maintenance and permeability. A 2020 systematic review on the Tie2 signaling pathway emphasized its therapeutic potential for retinal vascular diseases [15]. (3) Abnormal regulation of endogenous antiangiogenic proteins. Broad-spectrum antiangiogenic treatments have been shown to be effective for ocular neovascular diseases since 2010 [16]. Therefore, neovascularization regulation factors, the Tie2 pathway, and endogenous antiangiogenic proteins are not only major components of CNV pathogenesis but also potential therapeutic targets for CNV diseases.

Although various potential biomarkers have been identified for CNV diagnosis or treatment, more therapeutic targets are still needed for effective interference regarding CNV pathogenesis and CNV treatment. Computational approaches have also been introduced to find novel biomarkers or pathological mechanisms associated with CNV. The shortest path algorithm in protein–protein interaction networks at either the gene or function level has already been used to explore CNV pathogenesis [17,18]. Additionally, the Laplacian Heat Diffusion (Diffusr) algorithm has also been reported to be effective for novel CNV gene signature identification [19], implying the efficacy and accuracy for computational approaches use in CNV pathogenesis exploration.

Here, in this study, we introduce another method, GenePlexus 3.0.0, a web-based network-dependent gene similarity predictor, to explore novel gene signatures associated with CNV [20]. Our study not only provides a novel computational approach for disease biomarker identification but also identifies a series of novel disease gene signatures, helping to reveal the molecular mechanisms of CNV.

## 2. Materials and Methods

### 2.1. Choroidal Neovascularization-Associated Genes

Choroidal neovascularization is a complication usually associated with various retinal diseases, which involves the growth of new blood vessels originating from the choroid and extending into the subretinal space through the rupture of Bruch’s membrane. If not treated in time, this process will lead to serious vision loss. In order to explore the genetic basis of CNV, we used DisGeNET [21], which is a comprehensive database to compile gene–disease associations from sources including expert-managed databases, scientific documents, and electronic health records. This will help us to further study the genetic basis of CNV. By employing the keyword “choroidal neovascularization,” we identified a set of genes associated with this condition. Through DisGeNET, we discovered 33 genes linked to CNV, as shown in Table S1. These identified genes were then integrated into the PPI network.

The 33 seed genes should be interpreted as a non-etiology-stratified gene set associated with the DisGeNET disease concept “choroidal neovascularization.” The database query did not distinguish CNV secondary to nAMD from CNV associated with pathologic myopia, inflammatory chorioretinopathies, or other disorders. Accordingly, the seed set is neither an etiology-balanced representation of all forms of CNV nor an exclusively AMD-specific gene set. Because the amount of published and curated evidence differs among etiologies and is likely greatest for nAMD, an AMD-related evidence bias may be present. The downstream candidates are therefore interpreted as genes associated with the indexed CNV phenotype and require etiology-specific validation before being generalized to individual clinical disorders. The present DisGeNET query targeted the CNV disease concept and did not stratify the retrieved associations by type 1, type 2, or type 3 MNV; accordingly, the results should not be interpreted as a molecular analysis of all MNV subtypes, particularly retinal-origin type 3 lesions.

### 2.2. PPI Network

In this study, we focus on identifying proteins related to CNV and use GenePlexus within the framework of the PPI network. The PPI network is an important information type to accurately locate and predict essential genes, and it forms the basis of our exploration so that we can effectively use its comprehensive structure. This method allowed us to discover new candidate genes related to CNV, thus expanding our understanding of the genetic landscape beyond the existing findings.

The PPI data we studied were obtained from the STRING database (version 10, https://www.string-db.org/, accessed on 10 January 2024) [22], including 4,274,001 interactions among 19,247 human proteins, which are described in detail in the file “9606.protein.links.v10.txt.gz”. In this database, an interaction is described as a link between protein pairs, and each protein pair is identified by their Ensembl IDs. Each PPI is assigned a confidence score ranging from 1 to 999, which is derived from the combination of various sub-scores, including neighborhood, fusion, co-occurrence, co-expression, experimental evidence, database integration, and text mining analysis. Compared to PPI data collected in other databases, such as the Database of Interaction Proteins (DIP) [23] and BioGRID [24], which only report experimentally validated PPIs, PPIs in STRING provide a comprehensive assessment of protein interactions, taking into account various factors, such as genomic proximity, gene fusion events, cross-species incidence, co-expression patterns, and scientific literature confirmation.

In order to construct the PPI network, 19,247 human proteins were used as nodes. Connections (or edges) between these nodes were established based on the existence of PPIs between corresponding proteins, provided that they had confidence scores above zero. The strength of these interactions was expressed quantitatively in the edge weight, with a higher score indicating a stronger correlation. This method framework was helpful for the detailed and meticulous evaluation of protein interactions so as to identify the potential genetic markers of CNV more accurately.

### 2.3. GenePlexus

To tackle the challenge of pinpointing genes within molecular network G that are significantly correlated with the predefined group of genes S that are implicated in specific biological functions, pathologies, or phenotypes, Liu and his team devised the computational tool GenePlexus [25]. This sophisticated platform excels in evaluating gene relevance within molecular network G concerning the gene collection S. GenePlexus delineates the gene network G using three distinct matrices, namely, the adjacency matrix, the influence matrix, and the node embedding matrix. These matrices use the local structure of the network as an attribute, where each row represents a gene, and each column encapsulates a feature. For a specific gene set S, the constituent genes are treated as positive samples. By excluding the genes in these positive and S-like clusters, the residues in a large number of gene banks are classified as negative samples. Employing an elected representation matrix to encode these specimens supports the calibration of a logistic regression model embedded with L2 regularization. This model gauges the associative degree of each gene in network G to the gene set S. Advancing the work of GenePlexus, Mancuso and colleagues introduced PyGenePlexus (https://pypi.org/project/geneplexus/, accessed on 12 January 2024) [26], a Python-based software package that replicates the capabilities of its predecessor. PyGenePlexus endows users with the capacity to input gene batch S, specify gene topology G, and select a type of representation matrix, yielding outputs that encompass the significance of every gene within network G related to S (expressed as a calculated likelihood), interconnections among the most relevant genes, affiliated gene clusters (including recognized pathways, biological processes, and medical conditions), and fidelity of prediction (appraised by cross-validation techniques). Through a series of prediction tasks and strict comparisons of various gene networks, it has been proven that GenePlexus is superior to the traditional marker propagation method, which significantly enhances its utility in genome research [25]. Here, we input the PPI network and CNV-related gene set into PyGenePlexus. The output included the importance of each gene related to the CNV-related genome in the PPI network, which was expressed as a calculated probability. Then, we arranged the genes in descending order of probability to identify new genes related to CNV.

### 2.4. Screening Tests

Our findings showed that the specific candidate genes pinpointed by GenePlexus significantly correlated with the PPI network structure and potentially exhibited unique characteristics. For example, some nodes in the network may be classified as central nodes in advance, regardless of the seed nodes initially selected. By contrast, candidate genes that demonstrated denser linkages with genes previously confirmed were more frequently identified as novel genes associated with CNV. To enhance the precision in pinpointing these pivotal candidate genes, we devised three supplementary screening tests.

#### 2.4.1. Permutation Test

The configuration of the PPI network significantly affects the output of GenePlexus because some genes are likely to be highlighted based on their strategic position in the network. However, it must be admitted that not all these prominent genes are directly related to CNV. To discern the relevance of these genes, we utilized a permutation test that was initiated by generating 1000 arbitrary gene sets, labeled from *S*1 to *S*1000, each comprising a number of Ensembl gene identifiers equal to those linked with CNV. For each set, the included Ensembl IDs were analyzed as initial nodes in GenePlexus, assigning distinct probabilities to each gene. We then computed a *p*-value for each examined gene *g* using the equation:

(1) $$p_{value\left(g\right)}=\frac{N}{1000}$$

where N is the number of random groups whose GenePlexus scores exceed the true value. Genes with abnormally low *p*-values were therefore considered as new CNV-related genes because they rarely appear in random groups. We applied a *p*-value threshold of 0.05, which is the standard limit for reducing false positives in statistical tests. Therefore, genes below this limit were designated as potential candidate genes for follow-up research.

#### 2.4.2. Interaction Test

This study investigated the possible relationship between specific genes and CNV-related severe ocular disorders. This included analyzing the relationship between each problematic gene and those genes that have been confirmed to be related to CNV. A key index in this analysis was the strength of the protein interaction, which was quantified by the confidence score in the STRING database. For any specified gene, denoted as g, the methodology included calculating the maximum interaction score (MIS) it achieved with any protein, $g^{\prime }$, known to be associated with CNV, using the equation:

(2) $$MIS\left(g\right)=Max\left\{\begin{array}{c} Q\left(g,g^{\prime }\right):\;g^{\prime }\;is\;a\;validated\;\\ choroidal\;neovascularization\;associated\;gene \end{array}\right\}$$

where $Q\left(g,g^{\prime }\right)$ represents the confidence score between g and $g^{\prime }$ in STRING. Genes with high MIS values were considered to be more likely to contribute to CNV forms, reflecting the robustness of their protein–protein interactions. In order to identify the most promising genes, we set the minimum deletion threshold to 0.9, which is the cutoff of the highest confidence reported in STRING database. Interactions with confidence scores higher than this threshold occurred with a very high probability, suggesting a strong association between the two proteins in the interaction. Thus, this strict threshold ensured that only those genes that have a strong and conclusive relationship with CNV were selected.

#### 2.4.3. Enrichment Test

The purpose of this final analysis was to improve the selection of candidate genes by evaluating the degree of correspondence between the functional terms of candidate genes and those related to CNV. If the functional terms of a gene are very similar to those of genes that have been proven to play a role in CNV, then the applicability of this gene will be enhanced. This analogy was quantitatively evaluated by calculating the enrichment score, and the formula is as follows:

(3) ESg, F= −log10∑k=mnMkN−Mn−kNn

In this formula, F represents a specific GO term or KEGG pathway, *N* represents the total number of human genes, M represents genes marked by F, n represents the number of genes interacting with gene g according to the STRING database, and m represents how many of these interacting genes are also marked by F. For each gene g, we computed the 297 enrichment scores on 297 KEGG pathways and 20,681 enrichment scores on 20,681 GO terms, which were collected in vector V(g). Then, the relationship between any two genes g and $g^{\prime }$ was quantified by measuring the cosine similarity between their vectors:

(4) $$\Phi \left(g,\;g^{\prime }\right)=\frac{\;V\left(g\right)\cdot V\left(g^{\prime }\right)}{\left\|V\left(g\right)\right\|\cdot \left\|V\left(g^{\prime }\right)\right\|}$$

Furthermore, the maximum enrichment score (MES) for each candidate gene g was determined as follows:

(5) $$MES\left(g\right)=Max\left\{\begin{array}{c} \Phi \left(g,g^{\prime }\right):\;g^{\prime }\;is\;a\;validated\;\\ choroidal\;neovascularization\;associated\;gene \end{array}\right\}$$

In order to isolate the most promising candidates connected with CNV, we set the MES threshold to 0.95. The functional distribution map of the genes determined by this standard not only showed significant similarity with CNV-related genes but also demonstrated great potential correlations with CNV.

## 3. Results

**Figure 1:**
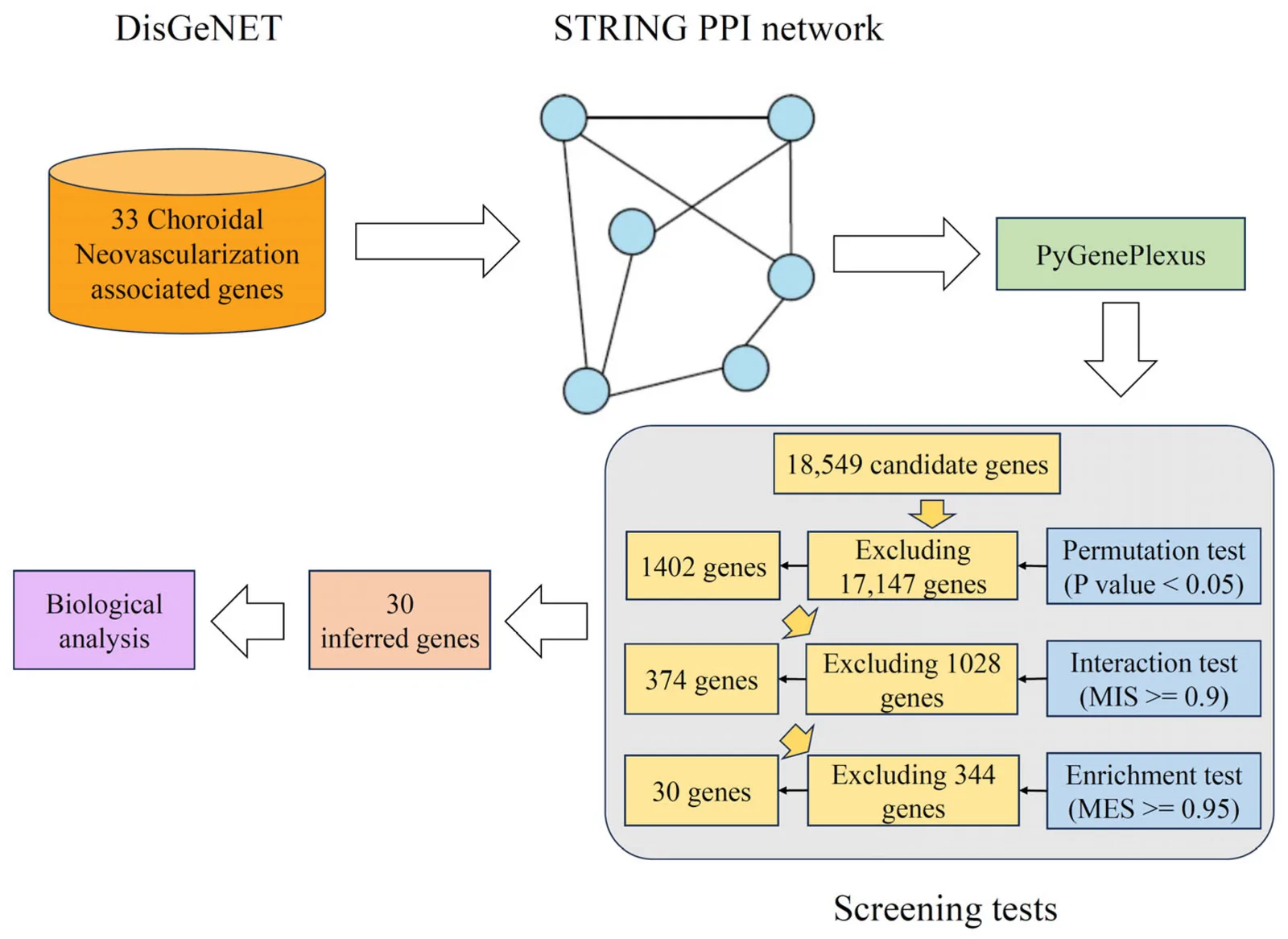
Flowchart depicting the procedure of extracting genes linked to choroidal neovascularization. Initially, we gathered validated genes associated with CNV from the DisGeNET database. Following this, we constructed a PPI network using data from the STRING database, a crucial step in visualizing the complex interplay between proteins. The GenePlexus tool was then utilized on this gene network to highlight candidates with a high likelihood of relevance. Subsequent refinement of these potential candidates was conducted through a series of screening tests, aiming to distill the list to a final set of genes deemed most pertinent. The culmination of our process involved a detailed evaluation of the relationship between these inferred genes and CNV, employing a literature-based analysis to substantiate their relevance to the disease.

This study utilized GenePlexus to identify new genes associated with CNV, as illustrated in Figure 1.

### 3.1. Results of the GenePlexus and Screening Tests

In this study, we analyzed the PPI network that targeted CNV-related proteins with GenePlexus. Initially identified as points of interest, these proteins were marked as “seed nodes.” We calculated the association probability of each network node with the seed node, excluding the seed node itself, and compiled these probabilities in Table S2. Then, we determined the protein corresponding to these specific nodes.

In order to determine the statistical significance of each protein, we conducted a permutation test to generate a *p*-value, as recorded in Table S2. Initially, proteins with a *p*-value less than 0.05 were considered important, which led to the discovery of 1402 proteins related to CNV. An additional review was conducted through the interaction test, which assigned an MIS value for each protein, as detailed in Table S2. Only proteins with an MIS value of 0.9 or above were selected, and there were 374 proteins related to CNV. In order to further explore their correlation, we carried out an enrichment test on these selected proteins, and determined the MES value of each protein, with the cutoff value set at 0.95. Proteins falling below this threshold were omitted, culminating in a concise roster of 30 significant proteins related to CNV, as delineated in Table S2. Referred to as ‘inferred proteins’ in subsequent analyses, these proteins were regarded as highly indicative of CNV associations.

### 3.2. Associations Between Inferred Proteins and Validated Proteins

In our research, we conducted a number of analyses to verify the reliability of forecasting proteins related to CNV. Specifically, we measured the number of interactions between each predicted protein and CNV-related genes in the PPI network, and we set the threshold of confidence as 0.9.

All inferred proteins were at least connected to one known CNV-associated protein. The linkage relationships between the inferred proteins in this study and the known CNV-associated proteins in STRING are provided in Table S3. Among these, THBS1, PDGFB, SERPINE1, and MMP2 are all connected to three known CNV-associated proteins, demonstrating their high correlation with CNV. By examining and comparing the connections between genes inferred from our study concerning CNV and their specific disease-related genes, we ascertained that the genes identified through our computational analysis were strongly connected to both conditions. This linkage indicates a probable role of these inferred genes in the development and progression of CNV, thereby underscoring the accuracy of our research methodology.

### 3.3. External Validation with Human Transcriptomic Data

**Figure 2:**
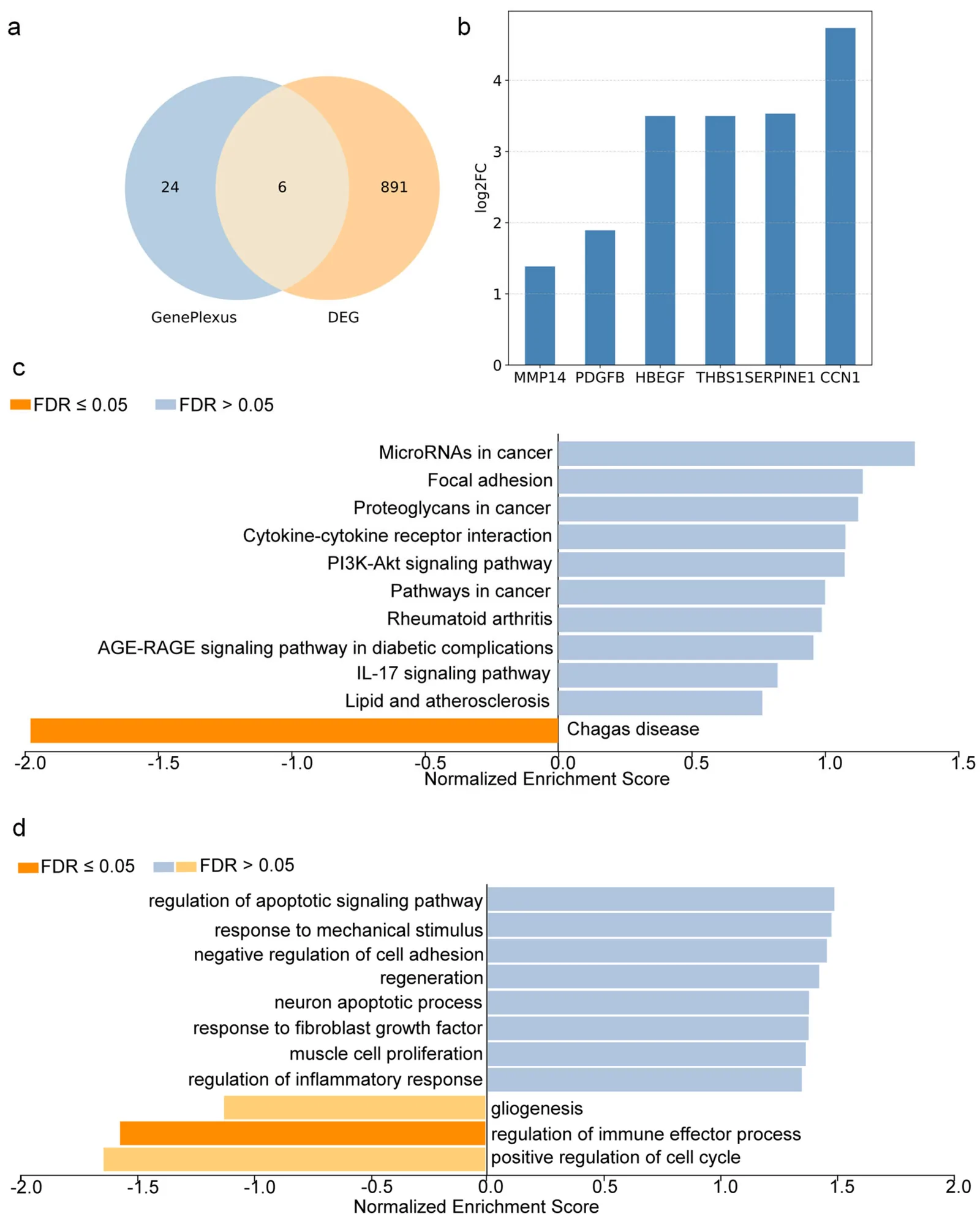
External validation and functional enrichment analysis of inferred genes. (**a**) Venn diagram illustrating the intersection between genes identified by GenePlexus and differentially expressed genes (DEGs) identified in human CNV membranes from an independent transcriptomic dataset. A total of six genes were found to be shared between the in silico predictions and the clinical data. (**b**) Bar chart displaying the log2 fold change of the six validated genes in the human CNV dataset. All genes showed statistically significant upregulation in disease tissues compared to controls. (**c**) Bar plot showing the top enriched KEGG pathways. (**d**) Bar plot showing the top enriched GO biological processes.

To validate the clinical relevance of the genes identified by GenePlexus, we analyzed an independent transcriptomic dataset derived from human CNV membranes [27]. This dataset compared the gene expression profiles of surgically excised human CNV membranes against healthy RPE-choroid control tissue. We extracted the differentially expressed genes (DEGs) from the human cohort using a significance threshold of *p* < 0.05. Intersecting these clinically derived DEGs with our top GenePlexus predictions yielded 6 common genes (MMP14, PDGFB, HBEGF, THBS1, SERPINE1, and CCN1), confirming that our network-based predictions captured actual transcriptional changes occurring in human pathology (Figure 2a). The expression levels (log2 Fold Change) of these validated genes are illustrated in Figure 2b. The consistency between our in silico predictions and the ex vivo human data reinforced the robustness of the GenePlexus algorithm in identifying biologically relevant disease signatures.

### 3.4. Literature-Based In Vivo Corroboration of Selected Candidates

To further assess the biological relevance of the computational results, we examined published gene-specific evidence from experimental CNV models. MMP2, one of the proteins prioritized in our analysis, has been investigated using mice deficient in MMP-2, MMP-9, or both enzymes. Following laser-induced rupture of Bruch’s membrane, combined MMP-2/MMP-9 deficiency markedly attenuated the incidence and severity of CNV, while endogenous or synthetic MMP inhibition also reduced the pathological response [28]. Independent in vivo evidence was also available for SERPINE1, which encodes plasminogen activator inhibitor-1 (PAI-1). In a laser-induced mouse model, PAI-1 deficiency prevented subretinal choroidal angiogenesis, whereas adenoviral restoration of PAI-1 expression recovered the wild-type angiogenic phenotype [29]. These loss-of-function and restoration experiments provide independent in vivo corroboration of the biological relevance of MMP2 and SERPINE1, two candidates recovered by our network-based analysis. They support the ability of the computational framework to prioritize genes with experimentally demonstrated roles in CNV, although they do not constitute prospective validation of the complete candidate set.

## 4. Discussion

**Figure 3:**
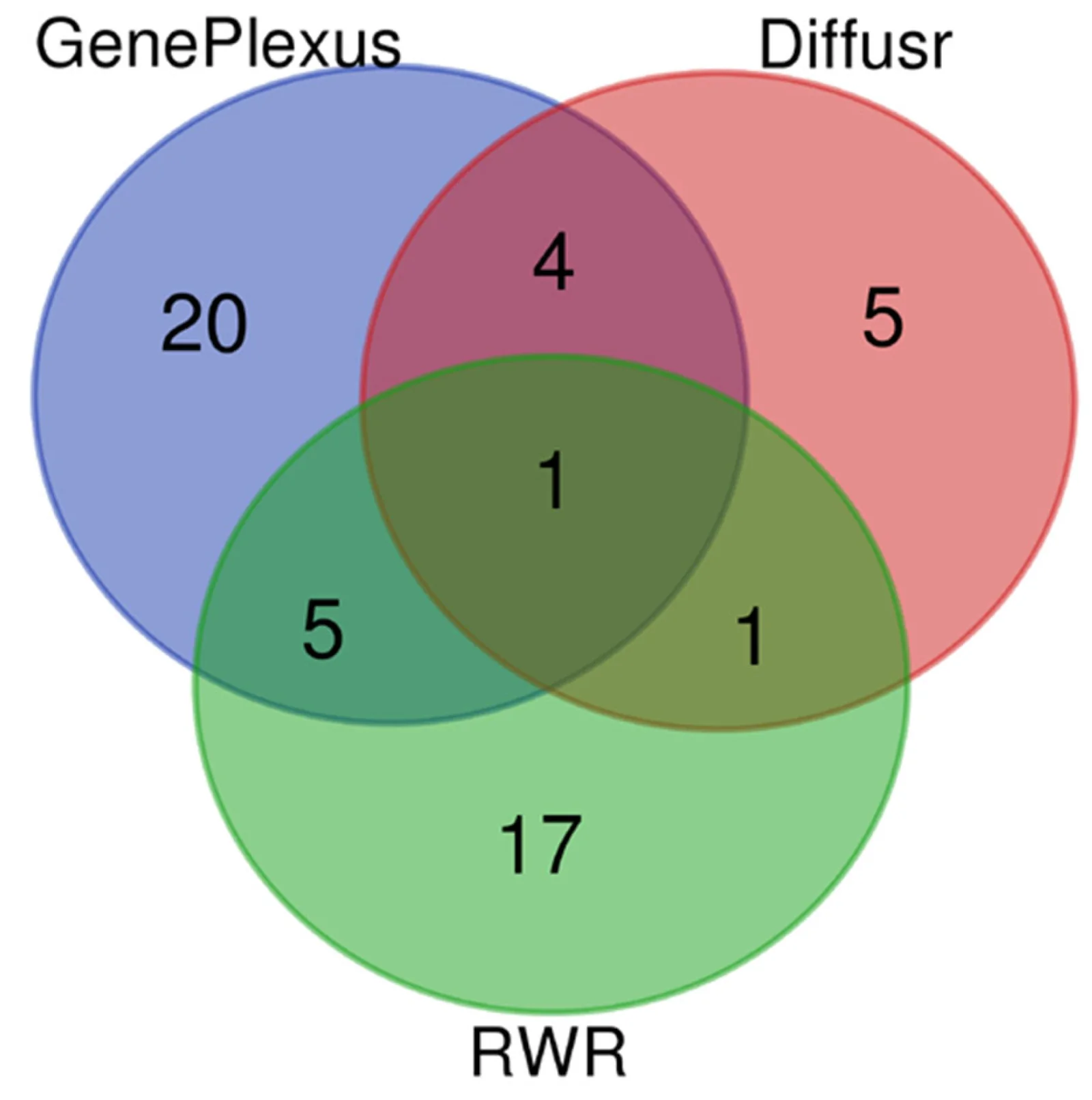
Venn diagram illustrating the overlap of CNV-associated genes as identified through the application of the Random Walk with Restart (RWR) method, the Heat Diffusion algorithm (Diffusr), and GenePlexus in our research.

In this study, we utilized the GenePlexus computational framework to expand the genetic landscape of CNV. Unlike traditional linkage analyses, our network-based approach identified a set of novel candidate genes that are topologically influential within the CNV interactome. Some important identified genes are listed in Table 1. We constructed a Venn diagram of CNV-related genes identified through Random Walk with Restart (RWR), Diffusr, and GenePlexus, which is shown in Figure 3. To validate these findings, we cross-referenced our candidates with external transcriptomic data from human CNV membranes, confirming the upregulation of key predicted genes in patient tissues. Furthermore, functional enrichment analysis of these candidates revealed that they do not act in isolation but rather form coordinated functional modules driving CNV progression (Figure 2c,d).

### 4.1. Pathway Enrichment Analysis of Identified Genes

Our KEGG pathway enrichment analysis identified several key signaling pathways significantly associated with the gene signature of CNV, providing mechanistic insight into the disease’s progression (Figure 2c). The PI3K–Akt signaling pathway emerged as a central hub in our analysis, consistent with literature confirming its pivotal role in hypoxia-induced neovascularization, where it stabilizes HIF-1α and subsequently upregulates VEGF expression in retinal pigment epithelial cells [30,31]. Furthermore, the enrichment of the focal adhesion pathway underscored the importance of cell–matrix interactions, as focal adhesion kinase (FAK) is essential for integrating signals from the extracellular matrix to regulate endothelial cell migration and proliferation during the invasive phase of CNV [32]. This interaction is physically mediated by structural proteins such as COL1A1 [33,34,35], while the necessary remodeling of the ECM is facilitated by the matrix metalloproteinases MMP2, MMP1, and MMP3 [36,37,38]. These enzymes cleave ECM components to enable signaling transduction and vascular sprouting, a process validated by their identification as top predicted targets in our analysis [39]. The broader role of extracellular matrix proteolysis in ocular neovascularization is reviewed in the context of inflammatory corneal neovascularization, where matrix remodeling regulates endothelial invasion and tissue repair [39]. These data provide independent support for linking the MMP candidates to extracellular matrix remodeling and vascular invasion in CNV [39]. We also identified the AGE–RAGE signaling pathway, which provides a molecular link between metabolic aging and inflammation; the accumulation of advanced glycation end products in drusen triggers oxidative stress via RAGE, promoting a pro-angiogenic environment [40]. Additionally, the IL-17 signaling pathway points to the immunovascular nature of the disease, where IL-17A can polarize macrophages toward a pro-angiogenic phenotype and stimulate VEGF production [41]. Finally, the association with lipid and atherosclerosis pathways aligns with the “lipid hypothesis” of AMD, suggesting that dysregulated lipid metabolism and macrophage recruitment to lipid deposits in Bruch’s membrane share mechanistic similarities with atherosclerotic plaque formation, driving the vessel growth observed in CNV [42]. This connection is reinforced by the identification of SERPINE1, F2 (Thrombin), and THBS1 [43,44,45]. As key regulators of fibrinolysis and coagulation, these genes highlight the contribution of thrombotic processes and plasminogen activation to the structural evolution of choroidal neovascular lesions.

### 4.2. Angiogenic Signaling via the PI3K–Akt Pathway

The gene ontology (GO) biological process analysis further dissected the functional roles of the identified genes, revealing a coordinated program of tissue remodeling and cellular response (Figure 2d). The prominence of response to mechanical stimulus highlights the physical aspect of CNV, where the rupture of Bruch’s membrane and the resulting mechanical stress on the RPE/choroid complex trigger mechanotransduction pathways essential for the initial sprouting of vessels [46]. Concurrently, the regulation of apoptotic signaling pathway suggests a dual role in the disease, regulating the delicate balance between the death of RPE cells—which creates a permissive environment for invasion—and the survival of endothelial cells within the neovascular complex [47]. The analysis also identified response to fibroblast growth factor (FGF), a critical mediator of the fibrotic scarring seen in late-stage CNV, where FGF signaling works synergistically with VEGF to drive the proliferation of fibroblasts and endothelial cells during the wound-healing-like response [48]. These processes are underpinned by the regulation of inflammatory response, confirming that our gene set captured the chronic inflammatory state of the AMD retina, where the recruitment of immune cells actively fuels pathological angiogenesis through cytokine secretion [49]. Central to this process is ICAM1, a predicted downstream effector of retinal inflammation and the receptor-associated prorenin system, which likely facilitates the leukocyte adhesion and infiltration necessary to sustain this chronic inflammatory state [50,51].

### 4.3. Top Genes Compared with Those Identified by Other Network Algorithms

Here, we compare our prediction results with previously reported results on CNV using other methods like Diffusr [19] and RWR. Compared to the Diffusr method, both methods identified various members of the matrix metalloproteinase family, like MMP3, implying the specific role of this protein family during the pathogenesis of CNV in the eyes. Apart from matrix metalloproteinases, both methods also identified collagen-related genes like COL1A1 and COL3A1. The functional relationship between abnormal collagen metabolism and CNV further validates the efficacy and accuracy of both prediction methods. As for RWR, we also identified collagen-related biomarkers like COL10A1 and COL8A1 in the top predicted gene list, validating the consistency. Therefore, previous methods like Diffusr and RWR also captured specific pathological mechanisms for CNV pathogenesis, while the newly presented GenePlexus-based approach identified more disease molecular signatures. The combined use of multiple machine learning approaches can provide a comprehensive understanding on the pathogenesis of complex diseases like CNV.

The comparison with previous methods clarifies why GenePlexus identified these specific modules. Traditional algorithms like RWR or Diffusr rely heavily on direct neighbor interactions. Consequently, they tend to find genes that are “popular” hubs or directly touch known bait genes. By contrast, GenePlexus uses node embedding to learn the “latent structural features” of a gene’s position. This allowed it to identify genes like SERPINE1 or COL1A1, which may not be direct neighbors of a specific bait gene but occupy a similar functional role within the network topology. COL1A1 has been reported to be associated with CNV at both the genetic [35] and transcriptomic levels [33]. Abnormal regulation of collagen has been widely reported during the initiation and progression of CNV [52], consistent with our identification.

Genes like F2, THBS1, and PDGFB were also only identified by GenePlexus. Thrombin has been shown to be functionally associated with the neovascularization process in the eyes and selected as potential therapeutic targets sensitive to photodynamic therapy [45]. Additionally, another gene in our prediction list, PDGFB, is a platelet-derived growth factor confirmed to be downstream of THBS1, which also participates in the neovascularization process in the eyes [53,54,55], validating the efficacy and accuracy of our identification. This capability enables the discovery of functionally related modules—such as the focal adhesion complex—that might be missed by simple distance-based methods.

All in all, as we have discussed above, we identified a series a potential CNV-associated genes with reliable literature support. Compared to previous RWR or Diffusr based analysis, we not only validated previous results but also identified a series of novel disease biomarkers, implying the efficacy of our newly presented method, GenePlexus.

### 4.4. Therapeutic Implications

Clinically available pharmacotherapy for CNV secondary to nAMD is dominated by intravitreal inhibition of vascular endothelial growth factor (VEGF). Ranibizumab and aflibercept inhibit VEGF signaling, bevacizumab is widely used off-label in many settings, brolucizumab is another anti-VEGF agent, and faricimab is a bispecific antibody that inhibits both VEGF-A and angiopoietin-2 [56]. Phase 3 trials have also established extended treatment intervals for faricimab and 8 mg aflibercept [57,58]. The Ang/Tie pathway provides a pathway-level connection between faricimab and our findings: faricimab inhibits angiopoietin-2, whereas our analysis prioritized ANGPT1, a Tie2 agonist involved in vascular stability. This relationship does not imply that faricimab directly targets ANGPT1 [59].

The functional clustering of these genes offers concrete therapeutic avenues beyond anti-VEGF monotherapy. Since the PI3K–Akt pathway integrates signals from multiple growth factors (VEGF, PDGF, and ANGPT1), targeting this central node could effectively block the “survival signal” for neovascular membranes. This represents a promising strategy for patients who are non-responders to anti-VEGF therapy, as it targets the intracellular convergence point of multiple angiogenic stimuli. Recent studies confirm that upstream regulators, such as the double-stranded RNA-activated protein kinase (PKR), actively drive CNV formation specifically through the PI3K/Akt axis, suggesting that blockade of this pathway could circumvent resistance mechanisms inherent to single-target therapies [60].

Our identification of MMP2, MMP1, and MMP3 highlights the critical role of extracellular matrix (ECM) degradation in the early invasive phase of CNV. Therapeutic strategies that inhibit these matrix metalloproteinases could physically prevent the rupture of Bruch’s membrane, a necessary step for choroidal vessels to invade the subretinal space. Historical data support this approach, showing that agents with MMP-inhibitory properties, such as doxycycline, can effectively attenuate the proteolytic environment required for lesion expansion [61]. Furthermore, the specific synergy between MMP-2 and MMP-9 in promoting angiogenesis suggests that selective gelatinase inhibition could serve as a maintenance therapy to prevent the reactivation of quiescent lesions [28].

Finally, the identification of SERPINE1 (PAI-1) and ICAM1 reveals a distinct therapeutic target at the intersection of coagulation and inflammation. Elevated levels of PAI-1 have been causally linked to pathologic angiogenesis and fibrosis, and genetic variants in SERPINE1 are associated with poor responsiveness to anti-VEGF agents. Inhibiting this axis offers a way to reduce the leukocyte infiltration mediated by ICAM1 while simultaneously checking the pro-angiogenic activity of the fibrinolytic system [62]. This “multi-hit” approach—targeting the inflammatory soil rather than just the vascular seed—aligns with recent findings that PAI-1 deficiency or blockade prevents the development of experimental choroidal neovascularization [29].

Taken together, currently available drugs primarily target extracellular VEGF or VEGF/angiopoietin-2 signaling, whereas direct inhibition of PI3K–Akt, MMP2/MMP1/MMP3, or SERPINE1/PAI-1 remains investigational in CNV. The preclinical effects of doxycycline and the experimental evidence implicating MMP-2 and MMP-9 support further target-validation studies but do not establish MMP inhibition as a clinical maintenance therapy [61]. Accordingly, the network-derived genes should be regarded as candidates for experimental validation rather than immediately actionable drug targets; their therapeutic value, ocular delivery, and safety require testing in appropriate experimental systems [63,64,65].

## 5. Conclusions

This research proves the effectiveness and adaptability of our gene discovery method, which combines DisGeNET data with the GenePlexus framework to identify key genes related to CNV. The method not only enriches the known CNV gene map but also reveals new genes that may be crucial to its pathogenesis. Key genes such as MMP2 and SERPINE1 play important roles in extracellular matrix degradation and plasminogen activation system, respectively. Their identification emphasizes the potential of our method for analyzing complex biological pathways. In view of the success of this method in describing the genetic factors of CNV, it is expected to be more widely used in gene discovery for various other diseases. By providing a powerful framework for identifying disease-related genes, our method can significantly enhance the understanding of the genetic basis of other complex diseases, thus promoting the development of targeted therapy and improving diagnostic accuracy. This generalization, coupled with its proven effectiveness, makes our method a valuable tool in the continuous exploration of understanding and fighting multifactorial diseases at the gene level. However, false-positive discoveries are inevitable given that we adopted computational methods to discover latent CNV-related genes rather than performing solid wet experiments. The genes identified in this study still need to be validated experimentally. The key code of this study is provided in File S1.

## Figures and Tables

**Table 1:** Important identified CNV-related genes.

Entrez	Symbol	Name
4313	MMP2	matrix metallopeptidase 2
5054	SERPINE1 ^$^	serpin family E member 1
4312	MMP1	matrix metallopeptidase 1
3383	ICAM1	intercellular adhesion molecule 1
4314	MMP3	matrix metallopeptidase 3
284	ANGPT1 ^$^	angiopoietin 1
2147	F2 ^$^	coagulation factor II, thrombin
7057	THBS1 ^$^	thrombospondin 1
1277	COL1A1 ^$^	collagen type I alpha 1 chain
5155	PDGFB ^$^	platelet-derived growth factor subunit B

^$^: Genes exclusively identified by our network-based method.

## Data Availability

Data are contained within the article or Supplementary Materials.

## Supplementary Materials

The following supporting information can be downloaded at: https://www.mdpi.com/article/10.3390/life16081263/s1, Table S1: Listing of the 33 gene IDs associated with CNV; Table S2: Detailed results of screening tests, including each filtering condition, for genes associated with CNV, presented in the corresponding sheet; Table S3: The linkages between the inferred proteins in this study and the known CNV-associated proteins in STRING; File S1: PyGenePlexus package and its input.

## Abbreviations

The following abbreviations are used in this manuscript:

CNV	Choroidal neovascularization
AMD	Age-related macular degeneration
DIP	Database of Interaction Proteins
MIS	Maximum interaction score
MES	Maximum enrichment score
DEG	Differentially expressed gene
RWR	Random Walk with Restart
FAK	Focal adhesion kinase
GO	Gene ontology
FGF	Fibroblast growth factor
