# Supplementary material for: Identifying Choroidal Neovascularization-Associated Genes with GenePlexus in a Protein–Protein Interaction Network
Source: Life (Basel). 2026 Jul 30;16(8):1263. doi: 10.3390/life16081263 (PMC13514973; doi:10.3390/life16081263)
Supplement: Supplementary file 1 [file life-16-01263-s001.zip › File S1.pdf]

File S1. PyGenePlexus package and its input.

The codes of PyGenePlexus package can be accessed at https://github.com/krishnanlab/PyGenePlexus.

The input of the PyGenePlexus package is as follows, which is the IDs of known CNV-related genes.

1191
1381
1409
1415
1421
1490
1524
2171
2202
2266
2335
2487
2670
3122
3309
348
4060
4239
4318
51056
6010
6017
6678
6876
7018
7076
7412
7431
7450
7458
7869
83483
102724652
